# Supplementary figures and images for: Correlations between personality traits and specific groups of alpha waves in the human EEG
Source: PeerJ. 2016 Jul 19;4:e2245. doi: 10.7717/peerj.2245 (PMC4957988; doi:10.7717/peerj.2245)

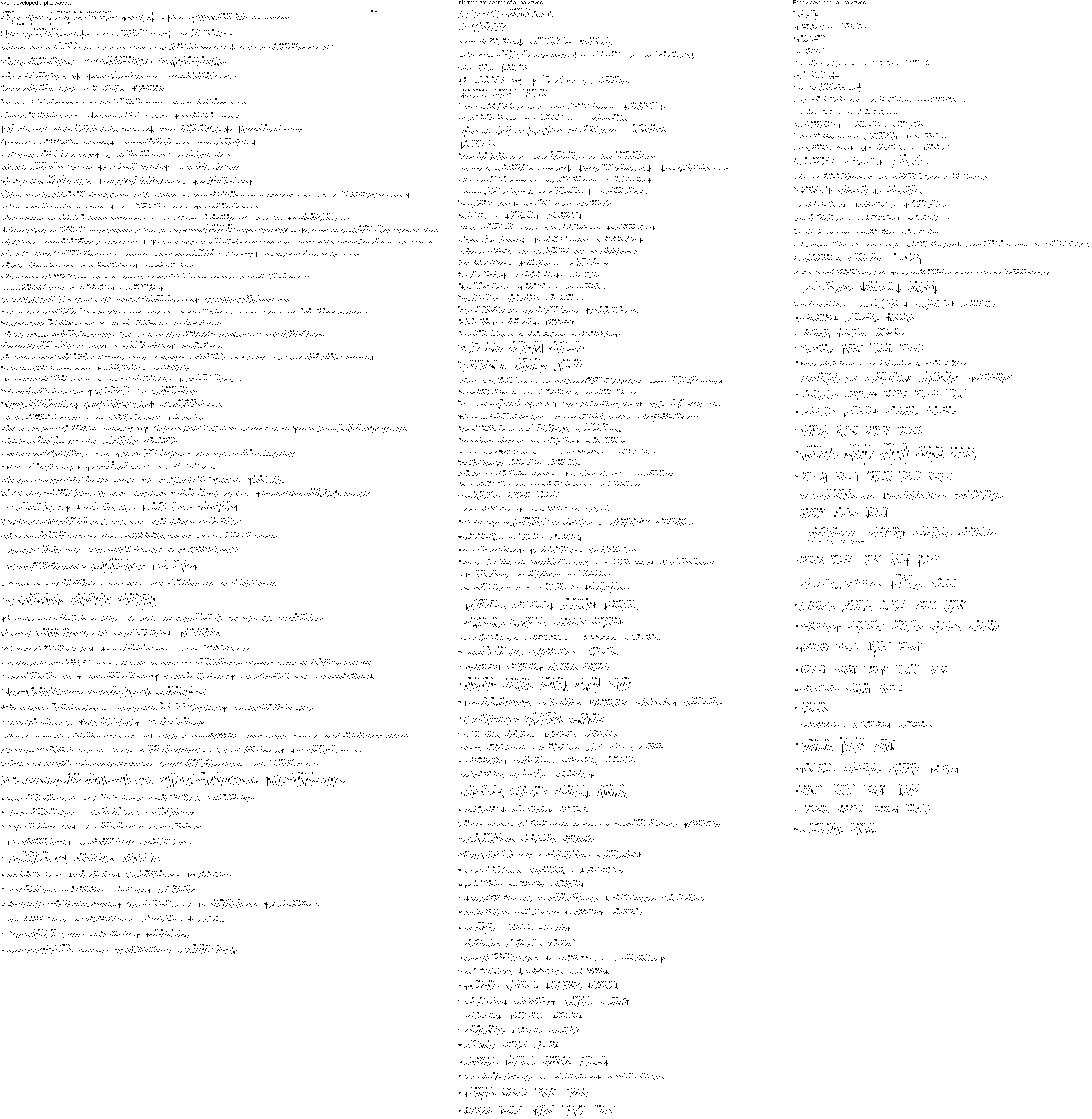

Supplement: Data S1 [file peerj-04-2245-s001.png]
